# Methylene Blue-Loaded Dissolving Microneedles: Potential Use in Photodynamic Antimicrobial Chemotherapy of Infected Wounds

**DOI:** 10.3390/pharmaceutics7040397

**Published:** 2015-09-28

**Authors:** Ester Caffarel-Salvador, Mary-Carmel Kearney, Rachel Mairs, Luigi Gallo, Sarah A. Stewart, Aaron J. Brady, Ryan F. Donnelly

**Affiliations:** School of Pharmacy, Queen’s University Belfast, Belfast, Co. Antrim BT9 7BL, UK; E-Mails: ecaffarelsalvador01@qub.ac.uk (E.C.-S.); mkearney16@qub.ac.uk; (M.-C.K.); rmairs06@qub.ac.uk (R.M.); luigigallo95@gmail.com (L.G.); sstewart35@qub.ac.uk (S.A.S.); aaron.brady@qub.ac.uk (A.J.B.)

**Keywords:** microneedle, PACT, wound, photosensitiser

## Abstract

Photodynamic therapy involves delivery of a photosensitising drug that is activated by light of a specific wavelength, resulting in generation of highly reactive radicals. This activated species can cause destruction of targeted cells. Application of this process for treatment of microbial infections has been termed “photodynamic antimicrobial chemotherapy” (PACT). In the treatment of chronic wounds, the delivery of photosensitising agents is often impeded by the presence of a thick hyperkeratotic/necrotic tissue layer, reducing their therapeutic efficacy. Microneedles (MNs) are an emerging drug delivery technology that have been demonstrated to successfully penetrate the outer layers of the skin, whilst minimising damage to skin barrier function. Delivering photosensitising drugs using this platform has been demonstrated to have several advantages over conventional photodynamic therapy, such as, painless application, reduced erythema, enhanced cosmetic results and improved intradermal delivery. The aim of this study was to physically characterise dissolving MNs loaded with the photosensitising agent, methylene blue and assess their photodynamic antimicrobial activity. Dissolving MNs were fabricated from aqueous blends of Gantrez^®^ AN-139 co-polymer containing varying loadings of methylene blue. A height reduction of 29.8% was observed for MNs prepared from blends containing 0.5% *w*/*w* methylene blue following application of a total force of 70.56 N/array. A previously validated insertion test was used to assess the effect of drug loading on MN insertion into a wound model. *Staphylococcus aureus*, *Escherichia coli* and *Candida albicans* biofilms were incubated with various methylene blue concentrations within the range delivered by MNs *in vitro* (0.1–2.5 mg/mL) and either irradiated at 635 nm using a Paterson Lamp or subjected to a dark period. Microbial susceptibility to PACT was determined by assessing the total viable count. Kill rates of >96%, were achieved for *S. aureus* and >99% for *E. coli* and *C. albicans* with the combination of PACT and methylene blue concentrations between 0.1 and 2.5 mg/mL. A reduction in the colony count was also observed when incorporating the photosensitiser without irradiation, this reduction was more notable in *S. aureus* and *E. coli* strains than in *C. albicans*.

## 1. Introduction

Chronic wound management has become a pressing global health issue due to a combination of factors, including, increased incidence of chronic wounds, rising levels of resistance to conventional treatments and lack of viable delivery methods for antimicrobials [1]. In the US, around 6.5 million patients are affected by chronic wounds, and an excess of US$ 25 billion is spent annually on their treatment [2]. Hence, there is a real need for novel antimicrobial techniques and delivery devices. In wounds, the protective barrier of the skin is damaged and in some cases totally absent, thus allowing microbial penetration and colonisation. Proliferation of pathogenic microorganisms results in infection which impairs wound healing and, in the majority of cases, this wound colonisation is polymicrobial. These wounds may become chronic, with a huge impact on patient morbidity, as well as on the health service in terms of treatment complexity and cost [3]. If not treated quickly and effectively, these infections may progress into deeper tissues and can eventually result in a systemic infection *i.e.*, bacteraemia [3,4,5].

A significant factor impeding wound healing is the presence of bacteria in the form of a biofilm, in contrast to their more treatable planktonic form. Biofilms are single or multi-species communities of microbial cells living cooperatively attached to surfaces or to each other. Biofilms constitute a challenge to wound healing, as they protect the colonised bacteria from both the patient’s immune response and antibiotic therapy [6]. The biofilm constituents are phenotypically and genotypically different, leading to a heterogeneous and dynamic structure, recalcitrant to antibiotics and the body’s immune response.

Infected wounds can be treated systemically or locally. However, due to poor vascularisation of the majority of wounds, antimicrobial accumulation does not occur at the site of infection when administered systemically [7]. Local delivery of antimicrobials may often be favoured, as the agent can be delivered directly to the intended site resulting in decreased systemic toxicity, with side effects generally localised to the site of application. In addition, local delivery helps to minimise the likelihood of antimicrobial resistance by reducing exposure of the antimicrobial to only pathogens at the wound site [5,8]. Conventional topical antimicrobial treatments for infected wounds such as mupirocin, neomycin and fusidic acid have been widely used clinically [9]. Although effective and often superior to their systemic counterparts, they are not without other problems. Due to widespread prescribing of many of these treatments, antimicrobial resistance is increasing [10,11,12]. Further to this, conventional delivery systems, such as gels, dressings and creams often struggle to overcome the barrier posed by necrotic tissue and, as a result, there is minimal penetration of the antimicrobial to the wound bed [4,5]. Some chronic biofilm infections, for instance, catheter infections or osteomyelitis, can persist in an indefinite manner unless the infected material is removed [13]. While local delivery is the favoured approach, effective treatment has become much more difficult and new techniques are widely sought after.

One novel antimicrobial technique is the use of photodynamic therapy (PDT). PDT is widely used for the treatment of cancerous and pre-cancerous skin lesions and has been investigated for treatment of microbial infections, termed photodynamic antimicrobial chemotherapy (PACT). This treatment involves the production of highly reactive species when a photosensitiser is delivered to its site of action and subsequently irradiated. These generated radicals can react with various biological components and results in destruction of microbial cells [14,15,16,17,18]. Due to its non-specific action on multiple cellular targets, the development of resistance to PACT is not thought to occur [17]. A wide range of microorganisms has shown to be susceptible to a number of photosensitisers with Gram positive and Gram negative bacteria, fungi and protozoa all proving susceptible *in vitro*. Importantly, human cells are not destroyed by this treatment, as the drugs exhibit selectivity for microbial cells [15,16].

In PDT, the photosensitizer is administered orally or intravenously, leading to its subsequent accumulation in neoplastic tissue. This does not occur in chronic wounds however, due to their avascular nature and, as such, in PACT the photosensitiser must be delivered topically [14,15,18]. The majority of studies focus on the *in vitro* activity of PACT but, in order to move further clinically, a novel drug delivery system must be developed [16]. Ideally, the drug delivery device should minimise the time between application and irradiation, conform to the shape of the wound whilst maintaining structural integrity and overcome the barrier of necrotic tissue [9].

Delivery via microneedles (MNs) is an attractive method for local administration of antimicrobials to wounds considering that they have already shown to be effective for improved delivery of photosensitisers [19]. MNs are 25–2000 µm in length and are arranged in arrays which can contain up to 2000 needles per centimetre squared [19]. These micron sized needles allow the passage of materials across the *stratum corneum* for trans/intra-dermal delivery of pharmacological substances by the creation of aqueous channels [20,21,22,23]. MNs may prove effective in delivery of photosensitisers to wounds as the needle projections should overcome the barrier posed by necrotic tissue in the same way, thus allowing delivery directly to the intended site of action [24]. Further to this, the incubation time between topical application of the photosensitiser and irradiation by light can be minimised since diffusional time may be reduced.

**Figure 1:**
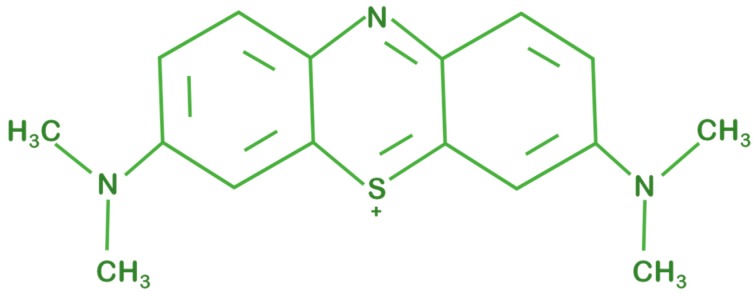
Methylene blue chemical structure.

Methylene blue (Figure 1) is a cationic preformed photosensitiser with numerous applications in PDT such as treatment of basal cell carcinoma and Kaposi’s sarcoma [25]. Methylene blue has also raised interest in its use for PACT [9]. Dissolving MNs are an ideal delivery system for this hydrophilic photosensitiser in PACT, as they should have the ability to cross the necrotic tissue and dissolve at the site of action to allow deposition of the drug molecule [22].

This study investigates the microbiocidal effect of methylene blue MNs against some common wound pathogens: *Staphylococcus aureus* (*S. aureus*), *Escherichia coli* (*E. coli*) and *Candida albicans* (*C. albicans*) in addition to testing the mechanical strength and insertion properties of methylene blue-loaded MNs. These MNs should exhibit sufficient mechanical strength to allow insertion into a wound site whilst releasing methylene blue efficiently and result in an antimicrobial effect after illumination.

## 2. Materials and Methods

### 2.1. Chemicals

Gantrez^®^ AN-139, a copolymer of methylvinylether and maleic anhydride (PVME/MA) was a gift from Ashland, Kidderminster, UK. Methylene blue was purchased from Sigma–Aldrich, Steinheim, Germany. Sodium hydroxide was obtained from Fisher Scientific UK Limited, Loughborough, Leicestershire, UK. Parafilm M™ was purchased from BRAND GMBH, Wertheim, Germany. All other chemicals used were of analytical reagent grade.

### 2.2. Microorganisms

*Staphylococcus aureus* ATCC 29213, *Escherichia coli* ATCC 25922 and *Candida albicans* NEYC 1467 were obtained from LGC standards, Middlesex, UK.

### 2.3. Preparation of Dissolving MNs Containing Methylene Blue

A 30% *w*/*w* aqueous solution of Gantrez^®^ AN-139 was adjusted to pH 7 by the addition of crushed sodium hydroxide pellets and monitored using a pH meter (HANNA instruments pH 209, Bedfordshire, UK). Blends containing 20% *w*/*w* Gantrez^®^ AN-139 and different quantities of methylene blue, 0.5% and 5%, were prepared. Approximately 500 mg of these formulations was added to laser engineered 14 × 14 silicon MN moulds, prepared as previously described [26], with a needle height of 600 µm. Moulds were centrifuged for 15 min at 3500 rpm (Eppendorf Centrifuge 5804, Davidson & Hardy Ltd., Belfast, UK) and left for 48 h at room temperature to dry after which arrays were extracted from the moulds and side-walls removed using a heated blade.

### 2.4. MN Compressibility Testing

Mechanical properties of MN arrays were investigated using a Texture Analyser (TA-XT2 Texture Analyser, Stable Microsystems, Haslemere, UK). The arrays were visualised using a light microscope, Leica EZ4 D digital microscope (Leica, Wetzlar, Germany) and needle height was measured using the ruler function. Using double sided adhesive tape, the MN array was attached to a probe which was moved by the Texture Analyser. The total force applied to 14 × 14 MN arrays was 70.56 N, corresponding to a force of 0.36 N per needle, pressed into a flat aluminum block. The probe was lowered at a speed of 0.5 mm/s until the target force was reached and the force was maintained for 30 s, with a post-speed of 1 mm/s. After the compression test, MNs were again visualised using the light microscope and MN heights measured.

### 2.5. Parafilm™ Insertion

MN insertion was assessed across Parafilm™, a model membrane used as an alternative to neonatal porcine skin for MN insertion testing, as previously described [27]. Briefly, eight layers of Parafilm™, with a thickness of approximately 1 mm, were stacked onto expanded poly(ethylene) for support. The MN array was inserted into the Parafilm™ using manual force for 30 s. The array was then removed and each layer of Parafilm™ peeled off with the number of holes in each layer visually determined using the Leica EZ4 D digital microscope. Parafilm™ sheets were placed between two polariser filters to facilitate easy detection of the created holes.

### 2.6. MN Permeation

Aluminum foil (approximately 0.02 mm in thickness) was used as an impermeable barrier to mimic the necrotic tissue that overlies many wounds. For this, MNs were applied with thumb pressure into aluminum foil mounted onto a modified Franz-cell setup. *In vitro* methylene blue permeation from MN arrays was studied using Franz cell receptor chambers (Crown Glass Co. Inc., Sommerville, NJ, USA) thermostated to 37 °C and containing phosphate buffered saline (PBS, pH 7.4). The orifice diameter of the donor and receptor compartments was 15 mm, with a permeation area of 0.25 cm^2^ corresponding to the area of the MN arrays with micro-projections. The volume of the receptor compartment was calculated to be 12 mL. Samples of methylene blue permeated were taken 5 and 30 min post-MN application and transferred to a 96-well plate for analysis.

### 2.7. Analysis of Methylene Blue Permeation

Three calibration curves were prepared daily for three consecutive days, using concentrations of methylene blue between 0–10 µg/mL in PBS, in order to validate the analysis method. Absorbance was determined using a UV microplate reader (Powerwave XS, Bio-Tek Instruments Inc., Minooski, VT, USA) at a wavelength of 664 nm and PBS was used as a blank. Medium samples collected at pre-established time points from the Franz-cell receptor chamber were analysed using this method.

### 2.8. Microorganism Susceptibility Testing and Photodynamic Therapy

*S. aureus* and *E. coli* were cultured into Mueller-Hinton broth (MHB) and placed in an orbital incubator at 37 °C. A sample was taken from the log phase and adjusted to an optical density at 550 nm equivalent to 1 × 10^8^ cfu/mL, using MHB in a sterile McCartney vial. *C. albicans* was cultured into Sabouraud Dextrose Broth (SDB) and placed into the incubator at 37 °C. Once in the log phase, the sample was adjusted to an optical density equivalent to 1~5 × 10^6^ cfu/mL at 530 nm [28], using SDB in a sterile McCartney vial.

To assess the susceptibility of biofilm-grown microorganisms to photodynamic killing, biofilm preparation was carried out by adding aliquots of 100 µL of the microorganism dilutions to a black-walled 96-well microtitre tray (Nalgen Nunc International, Rochester, NY, USA) and incubated for 24 h at 37 °C. Following incubation, the tray wells were gently washed three times with sterile PBS to remove any non-adherent microorganisms. Aliquots of 100 µL of either sterile PBS or sterile PBS containing a defined concentration of methylene blue was added at set times and incubated for 30 min [29]. The wells were then either irradiated (as described below) or subjected to a dark period for 5 min. Irradiation at 635 nm was performed using a Paterson Lamp (Phototherapeutics Ltd., Manchester, UK) with a fluence rate of 100 mW/cm^2^. Following incubation, wells were emptied, gently washed in PBS and 100 µL aliquot of sterile PBS was added. The plate was then sonicated for 10 min in a 150 W ultrasonic bath operating at a nominal frequency of 50 Hz. Serial tenfold dilutions in PBS were performed and three 20 µL drops of the different dilutions plated on Mueller-Hinton agar (MHA) or Sabouraud Dextrose agar (SDA). Following incubation for 24 h at 37 °C, total viable counts were determined [29].

### 2.9. Statistical Analysis

Where appropriate, statistical analyses to compare results were performed using a two-way analysis of variance (ANOVA). In all analyses, *p* < 0.05 denoted statistical significance. The Statistical Package GraphPad Prism^®^ Version 5.03 (GraphPad Software Inc., San Diego, CA, USA) was used for all statistical analysis.

## 3. Results and Discussion

PACT is an attractive antimicrobial technique for the treatment of chronic wounds, primarily due to the emergence of bacterial resistance to many conventional treatments. Resistance development against PACT has yet to be shown and, with numerous studies demonstrating *in vitro* killing of Gram-negative and Gram-positive bacteria, this may become an extremely valuable antimicrobial treatment [9,17]. For successful PACT treatment, the photosensitiser and light dose must be delivered directly to the wound. Delivery of photosensitisers to wounds is limited by various issues, namely the ineffectiveness of current topical delivery systems. Creams, gels and ointments are widely prescribed but do not overcome problems associated with wounds, such as the barrier posed by necrotic tissue [3]. Further to this, preformed photosensitisers tend to have large molecular weights and are hydrophilic in nature which results in formulation and penetration difficulties [19]. Enhanced delivery methods to wounds is therefore desirable. Dissolving MNs may offer a solution to this issue. MNs form channel-like pores in the *stratum corneum*, enabling delivery through this formidable barrier [23,30]. In the same way, these needles should cross necrotic tissue in wounds allowing local delivery of photosensitisers. The advantages offered are two-fold, as the MNs both overcome the necrotic barrier and facilitate delivery of the photosensitiser.

### 3.1. Compressibility Testing

**Figure 2:**
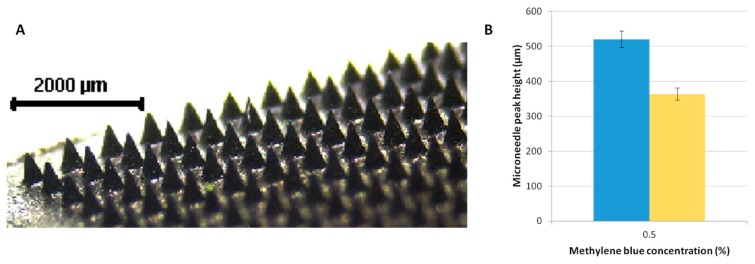
(**A**) Digital image of the methylene blue-loaded microneedle (MN). (**B**) Average microneedle peak heights before and after compression with a 70.56 N force applied to the array (Means ± S.D., *n* = 3).

Characterisation of MN mechanical properties is important to ascertain their proficiency in penetrating skin tissue [31,32]. MN compressibility is an indicator of physical integrity and thus by comparing this property at multiple drug loadings it can be deduced whether drug loading will have effect on MN strength. The MN array should retain its physical integrity to ensure full insertion and allow unhindered release of drug molecules. Figure 2A shows a 3D image of MNs containing methylene blue.

Figure 2B shows the average needle height before and after compression. The applied force caused a decrease in needle height of 29.8% in needles prepared from blends containing 0.5% *w*/*w* methylene blue. No results were obtained for 5% *w*/*w* methylene blue, as the needles shattered upon application to the aluminum block. This may indicate that increasing methylene blue loading above a certain point may decrease MN strength.

### 3.2. Parafilm™ Insertion

**Figure 3:**
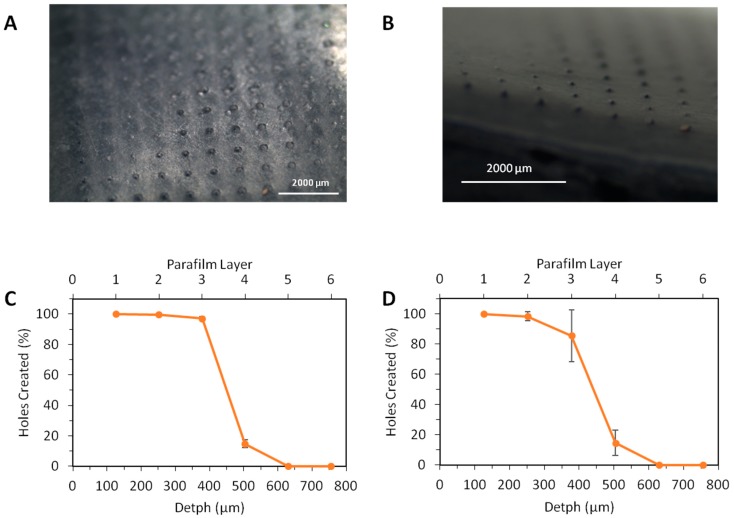
Image of (**A**) the Parafilm™ after microneedle insertion viewed using polarising lenses and (**B**) the microneedles piercing one layer of Parafilm™. Parafilm™ insertion test for (**C**) 0.5% and (**D**) 5% methylene blue loaded microneedles following a manual force application (Means ± S.D., *n* = 3).

MN insertion and thus tissue penetration is an important factor for successful drug delivery [27]. Accordingly, this experiment was used to determine whether 0.5% and 5% *w*/*w* methylene blue-loaded MNs are capable of insertion into human skin. Parafilm™ has previously been proven to act as a model mimicking human tissue in terms of MN insertion [27]. Figure 3A,B shows insertion of the MN through one layer of Parafilm™ as viewed under the digital microscope. It can be observed that almost full piercing of the array into the Parafilm™ occurs. Figure 3C,D shows the insertion profile of the MNs into the Parafilm™. It can be seen that insertion occurred to a greater degree in each of the first three layers and it can therefore be concluded that insertion of the both 0.5% and 5% *w*/*w* methylene blue occurs between Parafilm™ layer 3 and Parafilm™ layer 4, equating to distances between 378–504 µm. These results suggest that these MNs would have sufficient strength to penetrate through the *stratum corneum* and therefore also the layers of necrotic tissue in a wound.

The tested MNs did not break upon application to the Parafilm™ using a manual force. These results are promising. However, one drawback is the variability between patients in terms of manual insertion. Insertion forces can vary due to differences in weight, age and gender. This is an issue that will have to be addressed moving forward as assurance of dose uniformity will be necessary. Steps to address these issues have been taken with thorough patient counselling by a pharmacist on the application of MNs having been shown to increase reproducibility in terms of insertion depth and % needle insertion [33]. From this study, it can be concluded that insertion does successfully occur with both 0.5% and 5% *w*/*w* drug loaded MNs, using manual force, which will in all likelihood allow delivery of methylene blue across the necrotic tissue barrier.

### 3.3. In Vitro Permeation of Methylene Blue

Previous studies have demonstrated the successful delivery of small hydrophilic molecules such as methylene blue from soluble PMVE/MA MNs [34,35]. *In vitro* analysis using a modified Franz-cell setup was performed to investigate the concentration of methylene blue permeated from MNs. Table 1 shows the amount of methylene blue permeated.

**Table 1:** Methylene blue released by MN after 5 and 30 min of insertion across aluminum foil paper on a Franz cell setup (Means ± S.D., *n* = 3).

MN insertion time	Methylene blue permeated into the Franz cell (mg/mL) by MNs prepared from blends containing	
	0.05% methylene blue	5% methylene blue
5 min	0.01 ± 0.01	0.05 ± 0.02
30 min	0.03 ± 0.02	2.36 ± 0.97

Methylene blue concentrations covering the range between the minimum and maximum concentrations permeated from the MNs in the Franz-cell setup were selected to test in PACT. In particular, the minimum concentration of methylene blue permeated was found to be 0.1 mg/mL after 5 min application from MN prepared from a blend containing 0.05% of methylene blue, and the maximum concentration permeated was 2.36 mg/mL from MN prepared from a blend containing 5% of methylene blue after 30 min. Therefore, methylene blue concentrations of 0.1, 0.25 and 2.5 mg/mL were selected to conduct the microbiology studies. Lower methylene blue concentrations were not considered, as previous research demonstrated that bactericidal activity occurred using concentrations greater than 0.1 mg/mL [29,36].

### 3.4. Microorganism Susceptibility

Aerobic microorganisms, such as *Staphylococci* and *Micrococcus* species and anaerobic species such as *Propionibacterium* and *Acinetobacter* are examples of bacteria that colonise the skin, even under normal conditions. An equilibrium exists between the natural flora and the defences of the host, ensuring that bacteria do not uncontrollably proliferate. The reduction of these defences however, for instance in the case of an open wound, increase the likelihood of an infection [37].

It has previously been shown that bacterial kill is proportional to intensity of light delivered [12], but if the light dose is too high, this can be harmful to human tissue [38]. Continuous or high radiation doses can induce skin changes, such as dryness, but also structural changes including oedema of collagen bundles and changes in the connective tissue. At excessive doses, the irradiation causes the excitation or ionization of electrons in cells and can result in cellular damage of vital cell structures, including DNA. Similarly, the healthy tissue, for instance, the tissue surrounding the targeted wound, could be damaged if it was continuously exposed to irradiation unnecessarily [39]. In this study, irradiation was performed by a Paterson lamp, fixed at an output of 635 nm. If this technique was to be used in clinical practice, these lamps are simple to use and widely available in the UK making treatment relatively easy and minimising costs compared to expensive lasers for a specific photosensitiser [9].

**Figure 4:**
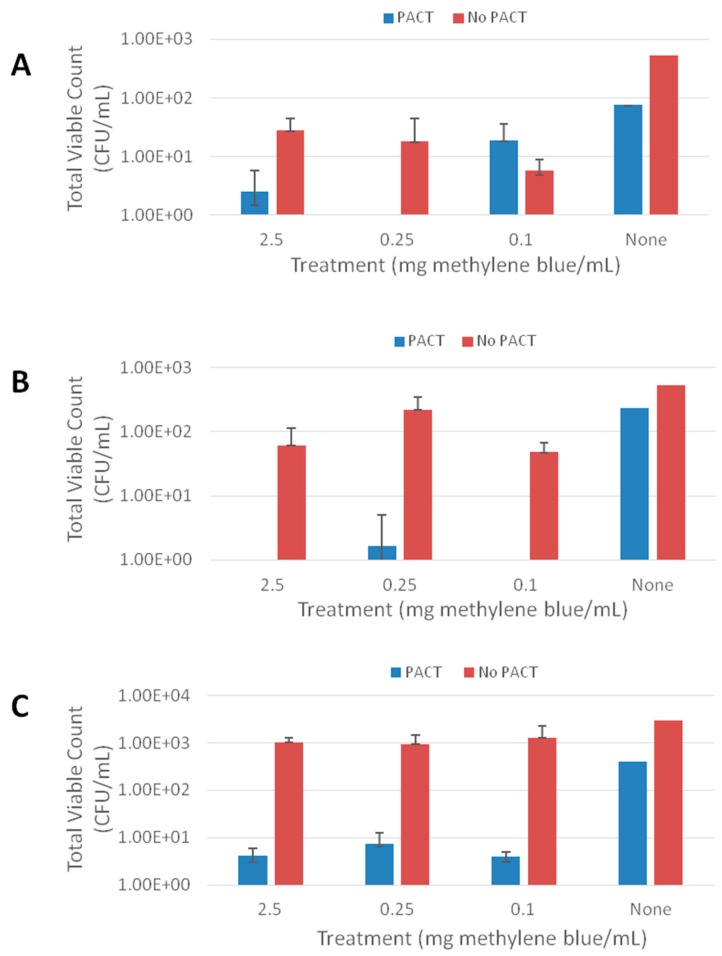
Effect of exposure to photodynamic antimicrobial chemotherapy (PACT) at a range of methylene blue concentrations on killing of biofilm grown strains (**A**) *S. aureus*, (**B**) *E. coli* and (**C**) *C. albicans* (Means ± S.D., *n* = 4).

The combination of methylene blue and PACT resulted in a significant reduction in viable count for each of the strains tested: *S. aureus*, *E. coli* and *C. albicans* (Figure 4). In particular, variations in the concentration of methylene blue resulted in a highly significant reduction in viable count for *S. aureus* (*p* < 0.0001) and *E. coli* (*p* < 0.0001) but did not significantly affect *C. albicans* (*p* = 0.2009). The application of PACT was significant for the strains, *p* values of 0.0004, <0.0001 and 0.0053 were obtained for *S. aureus*, *E. coli* and *C. albicans*, respectively. After the incubation of the different strains for 24 h, surviving organisms were compared to the negative control (no methylene blue and no irradiation). In all cases, the total viable count of the colonies not exposed to irradiation was at least a log_10_ greater than the counts of the wells irradiated for 5 min. Similar results were obtained by Vilela *et al.* (2012), where microbial reductions of 0.8–1.0 log_10_ were obtained with methylene blue concentrations of approximately 0.1 mg/mL for both *S. aureus* (ATCC 6538) and *E. coli* (ATCC 25922) [36]. Another study conducted by Zolfaghari *et al.* (2009) reported 1.40 log_10_ and 1.15 log_10_ microbial reductions for excisional wounds and scarifications on rats, respectively, that were contaminated with methicillin-resistant *S. aureus* [40].

The mean percentage kill was calculated for the different strengths of methylene blue with colonies grown in the absence of irradiation and methylene blue used as the negative control (Table 2). Kill rates of over 96% were observed for all strains tested when combining irradiation and methylene blue. Irradiation, combined with methylene blue concentrations of 2.5, 0.25 and 0.1 mg/mL led to 100.0%, 99.7% and 100.0% *E. coli* kill, respectively. For *C. albicans*, the combined treatment led to a 3 log_10_ colony reduction. In most cases, methylene blue alone, without the irradiation step, did have an effect on the viability of biofilm-grown isolates. When methylene blue concentrations of 2.5, 0.25 and 0.1 mg/mL were incorporated with no irradiation step, a mean kill rate of 94.7%, 96.6% and 98.9% was achieved for *S. aureus* respectively (Table 2A). This is in keeping with other findings in the literature, for instance, Usacheva *et al.* (2001) reported dark toxicity of methylene blue against several pathogens including *S. aureus* [41]. In addition, Donnelly *et al.* (2009) reported similar findings whereby methylene blue displayed bactericidal activity, even in the absence of irradiation [9]. The mechanism of action is not fully understood but it has been hypothesised that it is associated with guanylate cyclase inhibition and oxidation of the coenzyme nicotinamide adenine dinucleotide [42]. For *E. Coli,* kill rates between 59.1% and 90.9% were observed with the different methylene blue concentrations without PACT (Table 2B). A lower kill was obtained for photosensitiser without irradiation for the *C. albicans* strain, with kill rates of 66.3%, 69.1% and 57.3% for concentrations of methylene blue of 2.5, 0.25 and 0.1 mg/mL (Table 2C). *C. albicans* grows as a biofilm on epithelial surfaces; the oropharyngeal and vaginal tract being the primary sites of growth. PACT would offer an alternative for treatment of such infections and considering that most chronic wounds are polymicrobial in nature, PACT is a superior method for complete biofilm eradication. These results correlate with other *in vitro* studies that have shown that *C. albicans* is susceptible to photodynamic therapy using various photosensitisers [43,44]. In future studies, it would be necessary to determine the minimum inhibitory and minimum bactericidal concentrations of methylene blue to allow effective treatment of colonised wounds, yet minimise damage to commensal bacteria. It is likely that in patients with chronic wounds, PACT would need to be applied multiple times in order to ensure complete eradication of colonised pathogens and the inability to develop resistance would enable this to happen effectively. The selectivity of the photosensitisers to microbial cells also ensures no damage to human cells upon successive treatments [15].

Some *in vivo* studies have been completed on topical drug delivery devices for PACT. Of these, the majority have investigated the delivery of photosensitisers as solutions, which creates problems due to lack of retention at the wound site and inaccurate dosing [15,38,40]. A slightly different approach investigated the use of photosensitiser loaded hydrogels that allowed successful drug release at the wound site and resulted in *in vivo* bacterial cell death after illumination [9]. This did not overcome the barrier posed by the necrotic tissue usually present in chronic wounds however, and in clinical practice such an approach would necessitate surgical debridement prior to application. MNs would remove this requirement as the needles are capable of penetrating the outer necrotic tissue to allow photosensitiser delivery directly to the wound. MNs are subsequently removed and the wound irradiated with a resultant antimicrobial effect. A notable consideration of PACT is that to date, the *in vivo* bactericidal performance of methylene blue is much less than the *in vitro* effect [14,40]. This may be due to the limited penetrative ability of the photosensitiser to the wound bed using conventional delivery methods and as such the use of MNs may offer substantial benefit. Furthermore, of the few *in vivo* studies performed, it was shown that PACT has a more rapid effect on the reduction of bacterial load compared to conventional topical antimicrobials [38]. PDT has also been shown to promote wound healing by up-regulating growth factor expression and so the application of PACT in wounds is interesting [45]. Unlike planktonic microorganism growth, bacterial cells in biofilms exhibit reduced susceptibility to antibiotics and often require several treatments due to the properties of the biofilm environment [46].

Table 2Percentage kill of the strains (**A**) *S. aureus*, (**B**) *E. coli* and (**C**) *C. albicans* (Means ± S.D., *n* = 4).

**(A):** 

Treatment	Methylene blue concentration (mg/mL)			
	2.5	0.25	0.1	None
PACT	99.5 ± 0.6	100.0 ± 0	96.4 ± 3.0	89.4 ± 8.9
No PACT	94.7 ± 3.2	96.6 ± 4.9	98.9 ± 0.6	0 ± 0

**(B):** 

Treatment	Methylene blue concentration (mg/mL)			
	2.5	0.25	0.1	None
PACT	100.0 ± 0	99.7 ± 0.6	100.0 ± 0	56.6 ± 14.0
No PACT	88.4 ± 9.8	59.1 ± 24.9	90.9 ± 3.4	0 ± 0

**(C):** 

Treatment	Methylene blue concentration (mg/mL)			
	2.5	0.25	0.1	None
PACT	99.9 ± 0.1	99.8 ± 0.2	99.9 ± 0	86.2 ± 9.9
No PACT	66.3 ± 9.2	69.1 ± 19.0	57.3 ± 34.9	0 ± 0

Intrinsic antimicrobial properties of MN formulations have been demonstrated in several papers. This combined with PACT, further demonstrates the promising nature of such a delivery platform. Studies have shown an antifungal effect of the biocompatible copolymer, Gantrez^®^ AN-139 and an antimicrobial effect of Gantrez^®^ AN-169 [47]. MNs evidently show promise for the treatment of infections by the delivery of antimicrobials, and in particular photosensitisers. This study has shown that PACT has good bactericidal activity against *S. aureus* and *E. coli* in addition to antifungal activity against *C. albicans.* MNs could, therefore, be used for the delivery of antimicrobial agents topically to wounds or compromised skin, and their combination with PACT could be a viable alternative to the current antimicrobial treatments.

## 4. Conclusions

The polymicrobial nature of infected wounds, the presence of devitalised tissue and the emergence of antimicrobial resistant microorganisms has created a major problem in the treatment of infected wounds. The many limitations of current treatment methods necessitate novel delivery approaches. MNs are an ideal drug delivery system for wounds as, in addition to overcoming the layers of dead cells, their application in PACT may counter the challenges currently faced with topical delivery of photosensitisers. This study has demonstrated, for the first time, that photosensitisers delivered via dissolving MNs, with subsequent irradiation, are capable of eradication of biofilm forms of the common wound pathogens tested, as well as having the appropriate physical properties for insertion into the skin. In a clinical setting, this delivery platform could potentially offer considerable benefit as application times would be greatly reduced, due to the rapidly dissolving nature of the MNs and hence delivery of the photosensitiser. In addition, due to the reduced likelihood of resistance development, repeated application should not be an issue. Moving forward, future studies will assess the *in vivo* viability of the combinatorial approach of dissolving MNs and PACT. Such studies will investigate both the antimicrobial effect of the therapy as well as ascertaining a toxicity profile against healthy cells. This study provides proof-of-concept evidence that MNs offer promise for enhanced antimicrobial treatment of wounds and with a vast array of photosensitisers and antimicrobials already available, this technology offers significant scope for further variations and advancement.
